# Hexa-μ_2_-bromido-μ_4_-oxo-tetra­kis[(nicotine)copper(II)]

**DOI:** 10.1107/S1600536808018473

**Published:** 2008-06-25

**Authors:** Zhengjing Jiang, Guodong Tang, Lude Lu

**Affiliations:** a Key Laboratory for Soft Chemistry and Functional Materials of the Ministry of Education, Nanjing University of Science and Technology, 200 Xiaolingwei, Nanjing 210094, Jiangsu, People’s Republic of China; b Department of Chemistry, Huaiyin Teachers College, Huai’an 223300, Jiangsu, People’s Republic of China

## Abstract

In the title compound, hexa-μ_2_-bromido-μ_4_-oxo-tetra­kis{[3-(1-methyl-2-pyrrolidin­yl)pyridine-κ*N*]copper(II)}, [Cu_4_Br_6_O(C_10_H_14_N_2_)_4_], the four Cu atoms are tetra­hedrally arranged around the O atom at the cluster center. The Cu and coordinated N atoms lie along directions which correspond to four of the eight threefold axial directions of a regular octa­hedron. Each Cu atom lies at the center of a trigonal bipyramid, with the O atom and the pyridine N atom of a nicotine ligand in the axial positions and three Br atoms in the equatorial positions. Average bond distances are: Cu—N = 1.979 (8), Cu—O = 1.931 (6), Cu—Br = 2.514 (14) and Cu⋯Cu = 3.154 (6) ÅÅ. The configuration of the nicotine ligands is that of the *trans* diastereomer. In addition, the crystal structure contains five intra­molecular C—H⋯Br hydrogen bonds, which determine (or support) the orientation of the nicotine mol­ecules relative to their three equatorial Br atoms. One of the nicotine mol­ecules has two C—H⋯Br contacts, while the other three nicotine mol­ecules show only one C—H⋯Br bond each. Two other inter­molecular C—H⋯Br hydrogen bonds connect the complex mol­ecules, forming ribbons which extend in the *b*- and *c*-axis directions.

## Related literature

For related literature, see: Udupa & Krebs (1980 ▶); Meyer *et al.* (2006 ▶); Haendler (1990 ▶).
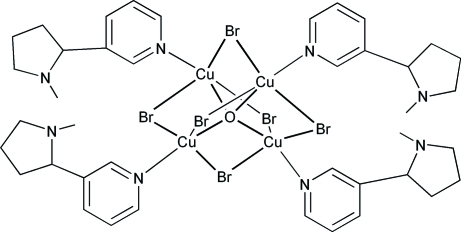


## Experimental

### 

#### Crystal data

[Cu_4_Br_6_O(C_10_H_14_N_2_)_4_]
*M* *_r_* = 1398.55
Monoclinic, 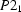

*a* = 12.9505 (5) Å
*b* = 13.2850 (3) Å
*c* = 14.2555 (2) Å
β = 92.221 (2)°
*V* = 2450.78 (11) Å^3^
*Z* = 2
Mo *K*α radiation
μ = 6.64 mm^−1^
*T* = 123 (2) K
0.20 × 0.16 × 0.14 mm

#### Data collection

Bruker SMART APEXII CCD diffractometer
Absorption correction: multi-scan (*SADABS*; Bruker, 2000 ▶) *T*_min_ = 0.29, *T*_max_ = 0.40
22605 measured reflections
9345 independent reflections
8124 reflections with *I* > 2σ(*I*)
*R*_int_ = 0.044

#### Refinement

*R*[*F* ^2^ > 2σ(*F* ^2^)] = 0.052
*wR*(*F* ^2^) = 0.122
*S* = 1.08
9345 reflections
536 parameters
1 restraint
H-atom parameters constrained
Δρ_max_ = 0.64 e Å^−3^
Δρ_min_ = −0.82 e Å^−3^
Absolute structure: Flack (1983 ▶), 4309 Friedel pairs
Flack parameter: 0.058 (15)

### 

Data collection: *APEX2* (Bruker, 2004 ▶); cell refinement: *SAINT* (Bruker, 2004 ▶); data reduction: *SAINT*; program(s) used to solve structure: *SHELXS97* (Sheldrick, 2008 ▶); program(s) used to refine structure: *SHELXL97* (Sheldrick, 2008 ▶); molecular graphics: *SHELXL97*; software used to prepare material for publication: *SHELXL97* and *PLATON* (Spek, 2003 ▶).

## Supplementary Material

Crystal structure: contains datablocks I, global. DOI: 10.1107/S1600536808018473/si2090sup1.cif

Structure factors: contains datablocks I. DOI: 10.1107/S1600536808018473/si2090Isup2.hkl

Additional supplementary materials:  crystallographic information; 3D view; checkCIF report

## Figures and Tables

**Table 1:** Hydrogen-bond geometry (Å, °)

*D*—H⋯*A*	*D*—H	H⋯*A*	*D*⋯*A*	*D*—H⋯*A*
C1—H1*A*⋯Br3	0.95	2.60	3.292 (9)	130
C15—H15*A*⋯Br4	0.95	2.71	3.362 (10)	126
C21—H21*A*⋯Br2	0.95	2.77	3.372 (10)	122
C25—H25*A*⋯Br6	0.95	2.75	3.332 (9)	120
C30—H30*C*⋯Br6^i^	0.98	2.92	3.844 (10)	158
C35—H35*A*⋯Br5	0.95	2.68	3.259 (10)	120
C39—H39*B*⋯Br5^ii^	0.99	2.88	3.764 (11)	150

Symmetry codes: (i) 
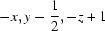
; (ii) 
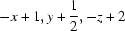
.

**Fig. 1.**
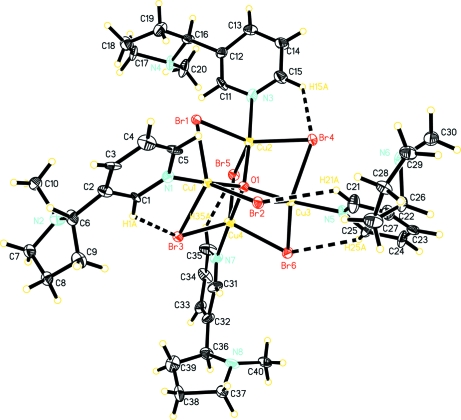
Molecular structure of the title compound, with atom labels and 30% probability displacement ellipsoids. Intramolecular hydrogen bonds are shown as dashed lines.

## supplementary crystallographic information

### Comment

Numerous clusters of nicotine [3-(1-methyl-2- pyrrolidinyl)pyridine] were reported to form molecular complexes with metals. But the crystal structures of the clusters containing both nicotine ligands and bromine atoms have not been reported so far. In order to explore the chemistry of nicotine clusters extensively, we synthesized the title cluster.

As illustrated in Fig. 1, the title compound has an O atom at the center of a tetrahedron of Cu atoms. The same O atom also lies at the center of a slightly distorted octahedron of Br atoms. This octahedron is in turn surrounded tetrahedrally by the four pyridine N atoms of the nicotine ligands, in parallel orientation with the Cu tetrahedron. The Cu atoms are bridged by the six Br atoms. The net effect is to place each Cu atom at the center of a slightly distorted trigonal bipyramid; the four bipyramids have six edges in common. The central O atom and the pyridine N atoms are in the axial positions, while the bridging Br atoms are in the equatorial positions. In addition, the absolut configurations of C6, C16, C26, and C36 can be given as S* (the * denotes unknown absolute configuration, but for the chosen coordinates the form appears to be S). The structure also contains five intramolecular and two intermolecular C—H···Br hydrogen bonds (Table 1). The intramolecular hydrogen bonds determine (or support) the orientation of the nicotine molecules relative to their equatorial three Br atoms. One of the nicotine molecules has two C—H···Br contacts: C21—H21A···Br2 and C25—H25A···Br6, the other three nicotine molecules show only one H bond. Two other intermolecular hydrogen bonds, C30—H30C···Br6 and C39—H39B···Br5, connect the complexes to form ribbons which extend in the *b* and *c* direction.

Examples of closely related compounds containing nicotine ligands include a mercury(II) chain polymer (Udupa & Krebs, 1980), a helical silver(I) coordination polymer (Meyer *et al.*, 2006) and a chloride-nicotine copper(II) complex (Haendler, 1990).

### Experimental

CuBr (1 mmol) was added to a solution of 4-cyanopyridine(1 mmol) in dmf (5 ml). The resulting mixture was stirred for about 10 min after which an orange precipitate formed. Nicotine (1 ml) was then added dropwise to the reaction mixture and stirring was continued, during which time the precipitate was dissolved, giving an orange solution.This solution then changed its colour to dark green with 30 min further stirring. The resulting solution was filtered and the dark green filtrate was transfered into a test tube and carefully laid on the surface of the filtrate with *i*-PrOH (10 ml). Dark-brown block crystals were obtained after 30 days. Yield: 0.158 g, 68% (based on CuBr used). Analysis: Found: C 34.52, H 3.90, N 7.90%; Calculated for C_40_H_56_Br_6_Cu_4_N_8_O: C 34.35, H 4.04, N 8.01%.

### Refinement

H atoms were positioned geometrically and refined using a riding model, with C—H = 0.95–1.00 Å and with *U*_iso_(H) = 1.2 (1.5 for methyl groups) times *U*_eq_(C). The Flack parameter used in the refinement is 0.058 (15) with 4309 Friedel pairs.

### Crystal data

**Table:**

[Cu_4_Br_6_O(C_10_H_14_N_2_)_4_]	*F*_000_ = 1372
*M**_r_* = 1398.55	*D*_x_ = 1.895 Mg m^−^^3^
Monoclinic, *P*2_1_	Mo *K*α radiation λ = 0.71073 Å
Hall symbol: P 2yb	Cell parameters from 8714 reflections
*a* = 12.9505 (5) Å	θ = 2.1–26.4º
*b* = 13.2850 (3) Å	µ = 6.64 mm^−^^1^
*c* = 14.2555 (2) Å	*T* = 123 (2) K
β = 92.221 (2)º	Block, dark brown
*V* = 2450.78 (11) Å^3^	0.20 × 0.16 × 0.14 mm
*Z* = 2	

### Data collection

**Table:**

Bruker SMART APEXII CCD diffractometer	9345 independent reflections
Radiation source: sealed tube	8124 reflections with *I* > 2σ(*I*)
Monochromator: graphite	*R*_int_ = 0.044
*T* = 123(2) K	θ_max_ = 26.0º
φ and ω scans	θ_min_ = 1.4º
Absorption correction: multi-scan(SADABS; Bruker, 2000)	*h* = −15→15
*T*_min_ = 0.29, *T*_max_ = 0.40	*k* = −16→16
22605 measured reflections	*l* = −17→17

### Refinement

**Table:**

Refinement on *F*^2^	Hydrogen site location: inferred from neighbouring sites
Least-squares matrix: full	H-atom parameters constrained
*R*[*F*^2^ > 2σ(*F*^2^)] = 0.052	*w* = 1/[σ^2^(*F*_o_^2^) + (0.07*P*)^2^ + 1.99*P*] where *P* = (*F*_o_^2^ + 2*F*_c_^2^)/3
*wR*(*F*^2^) = 0.123	(Δ/σ)_max_ < 0.001
*S* = 1.08	Δρ_max_ = 0.64 e Å^−^^3^
9345 reflections	Δρ_min_ = −0.82 e Å^−^^3^
536 parameters	Extinction correction: none
1 restraint	Absolute structure: Flack (1983), 4309 Friedel pairs
Primary atom site location: structure-invariant direct methods	Flack parameter: 0.058 (15)
Secondary atom site location: difference Fourier map	

### Special details

**Table:**

Geometry. All e.s.d.'s (except the e.s.d. in the dihedral angle between two l.s. planes) are estimated using the full covariance matrix. The cell e.s.d.'s are taken into account individually in the estimation of e.s.d.'s in distances, angles and torsion angles; correlations between e.s.d.'s in cell parameters are only used when they are defined by crystal symmetry. An approximate (isotropic) treatment of cell e.s.d.'s is used for estimating e.s.d.'s involving l.s. planes.
Refinement. Refinement of *F*^2^ against ALL reflections. The weighted *R*-factor *wR* and goodness of fit *S* are based on *F*^2^, conventional *R*-factors *R* are based on *F*, with *F* set to zero for negative *F*^2^. The threshold expression of *F*^2^ > σ(*F*^2^) is used only for calculating *R*-factors(gt) *etc*. and is not relevant to the choice of reflections for refinement. *R*-factors based on *F*^2^ are statistically about twice as large as those based on *F*, and *R*- factors based on ALL data will be even larger.

### Fractional atomic coordinates and isotropic or equivalent isotropic displacement parameters (Å^2^)

**Table:**

	*x*	*y*	*z*	*U*_iso_*/*U*_eq_	
Br1	0.28823 (7)	0.58585 (6)	0.76809 (6)	0.02612 (19)	
Br2	0.01897 (6)	0.79441 (7)	0.75355 (6)	0.0291 (2)	
Br3	0.25573 (7)	0.84149 (7)	0.95621 (6)	0.0305 (2)	
Br4	0.22450 (7)	0.78513 (7)	0.52996 (6)	0.02774 (19)	
Br5	0.48949 (6)	0.84084 (7)	0.72986 (6)	0.03022 (19)	
Br6	0.23762 (7)	1.04233 (7)	0.72339 (7)	0.0336 (2)	
C1	0.1183 (7)	0.6417 (7)	1.0069 (5)	0.0247 (18)	
H1A	0.1583	0.6947	1.0344	0.030*	
C2	0.0695 (7)	0.5719 (8)	1.0658 (7)	0.033 (2)	
C3	−0.0037 (10)	0.5038 (8)	1.0229 (8)	0.051 (3)	
H3A	−0.0480	0.4654	1.0606	0.061*	
C4	−0.0096 (8)	0.4941 (9)	0.9250 (8)	0.048 (3)	
H4A	−0.0492	0.4417	0.8960	0.057*	
C5	0.0429 (7)	0.5616 (7)	0.8717 (7)	0.030 (2)	
H5A	0.0341	0.5590	0.8053	0.036*	
C6	0.0778 (8)	0.5910 (8)	1.1674 (7)	0.041 (2)	
H6A	0.0488	0.5315	1.2004	0.049*	
C7	0.1780 (8)	0.6407 (9)	1.2997 (7)	0.041 (2)	
H7A	0.1709	0.5840	1.3441	0.049*	
H7B	0.2391	0.6815	1.3191	0.049*	
C8	0.0844 (7)	0.7018 (7)	1.2933 (6)	0.031 (2)	
H8A	0.1032	0.7739	1.2986	0.038*	
H8B	0.0400	0.6850	1.3461	0.038*	
C9	0.0258 (9)	0.6846 (9)	1.2030 (8)	0.046 (3)	
H9A	0.0332	0.7419	1.1594	0.055*	
H9B	−0.0485	0.6728	1.2130	0.055*	
C10	0.2290 (8)	0.5007 (8)	1.2051 (7)	0.038 (2)	
H10A	0.1901	0.4598	1.2487	0.057*	
H10B	0.3015	0.5043	1.2272	0.057*	
H10C	0.2247	0.4701	1.1425	0.057*	
C11	0.5028 (7)	0.5877 (8)	0.6410 (6)	0.035 (2)	
H11A	0.5097	0.5943	0.7073	0.042*	
C12	0.5629 (8)	0.5209 (7)	0.5960 (7)	0.036 (2)	
C13	0.5644 (7)	0.5044 (8)	0.4954 (7)	0.034 (2)	
H13A	0.6071	0.4565	0.4662	0.041*	
C14	0.4928 (8)	0.5699 (9)	0.4459 (7)	0.047 (3)	
H14A	0.4852	0.5675	0.3794	0.056*	
C15	0.4327 (8)	0.6395 (9)	0.4991 (7)	0.043 (3)	
H15A	0.3895	0.6853	0.4647	0.052*	
C16	0.6298 (7)	0.4506 (8)	0.6531 (7)	0.036 (2)	
H16A	0.6740	0.4120	0.6097	0.044*	
C17	0.7272 (11)	0.4266 (9)	0.7973 (8)	0.053 (3)	
H17A	0.7511	0.4612	0.8557	0.063*	
H17B	0.7824	0.3811	0.7766	0.063*	
C18	0.6280 (9)	0.3701 (8)	0.8109 (7)	0.046 (3)	
H18A	0.5875	0.4030	0.8598	0.056*	
H18B	0.6428	0.2999	0.8305	0.056*	
C19	0.5671 (9)	0.3718 (8)	0.7151 (8)	0.048 (3)	
H19A	0.4951	0.3946	0.7227	0.058*	
H19B	0.5660	0.3042	0.6858	0.058*	
C20	0.7870 (8)	0.5561 (10)	0.6860 (9)	0.053 (3)	
H20A	0.8067	0.5264	0.6264	0.080*	
H20B	0.8451	0.5513	0.7319	0.080*	
H20C	0.7687	0.6270	0.6762	0.080*	
C21	−0.0384 (8)	0.9135 (10)	0.5492 (8)	0.048 (3)	
H21A	−0.0547	0.8500	0.5757	0.057*	
C22	−0.1084 (9)	0.9577 (10)	0.4939 (7)	0.045 (3)	
C23	−0.0812 (7)	1.0480 (8)	0.4511 (7)	0.035 (2)	
H23A	−0.1274	1.0782	0.4060	0.042*	
C24	0.0026 (8)	1.0890 (9)	0.4715 (7)	0.045 (3)	
H24A	0.0172	1.1521	0.4435	0.054*	
C25	0.0777 (7)	1.0463 (8)	0.5346 (6)	0.033 (2)	
H25A	0.1406	1.0796	0.5515	0.039*	
C26	−0.2124 (9)	0.9171 (8)	0.4718 (7)	0.048 (3)	
H26A	−0.2532	0.9636	0.4294	0.058*	
C27	−0.2737 (10)	0.8910 (10)	0.5626 (9)	0.057 (3)	
H27A	−0.3146	0.9489	0.5844	0.068*	
H27B	−0.2275	0.8662	0.6145	0.068*	
C28	−0.3466 (7)	0.8022 (8)	0.5179 (7)	0.036 (2)	
H28A	−0.3459	0.7439	0.5612	0.043*	
H28B	−0.4186	0.8268	0.5107	0.043*	
C29	−0.3103 (8)	0.7697 (9)	0.4250 (8)	0.043 (2)	
H29A	−0.3540	0.7985	0.3732	0.051*	
H29B	−0.3104	0.6954	0.4194	0.051*	
C30	−0.1700 (9)	0.8187 (8)	0.3300 (8)	0.047 (3)	
H30A	−0.0999	0.8460	0.3263	0.070*	
H30B	−0.2188	0.8630	0.2957	0.070*	
H30C	−0.1726	0.7513	0.3020	0.070*	
C31	0.3732 (8)	1.0704 (7)	0.9282 (8)	0.039 (2)	
H31A	0.3001	1.0659	0.9290	0.047*	
C32	0.4159 (9)	1.1595 (8)	0.9843 (8)	0.044 (3)	
C33	0.5149 (8)	1.1545 (8)	0.9841 (8)	0.045 (3)	
H33	0.5517	1.1955	1.0286	0.054*	
C34	0.5729 (7)	1.0972 (10)	0.9273 (7)	0.045 (3)	
H34A	0.6423	1.1153	0.9164	0.054*	
C35	0.5316 (9)	1.0155 (9)	0.8872 (9)	0.052 (3)	
H35A	0.5729	0.9644	0.8610	0.063*	
C36	0.3478 (8)	1.2254 (9)	1.0366 (7)	0.041 (2)	
H36A	0.3857	1.2900	1.0485	0.049*	
C37	0.1705 (8)	1.2749 (8)	1.0538 (6)	0.038 (2)	
H37A	0.1051	1.2396	1.0367	0.045*	
H37B	0.1579	1.3483	1.0553	0.045*	
C38	0.2208 (9)	1.2342 (9)	1.1539 (7)	0.046 (3)	
H38A	0.2344	1.2908	1.1978	0.055*	
H38B	0.1740	1.1856	1.1834	0.055*	
C39	0.3163 (9)	1.1860 (9)	1.1298 (9)	0.051 (3)	
H39A	0.3065	1.1121	1.1263	0.061*	
H39B	0.3709	1.2005	1.1784	0.061*	
C40	0.2600 (8)	1.3053 (9)	0.9133 (7)	0.042 (2)	
H40A	0.3136	1.2742	0.8763	0.062*	
H40B	0.2805	1.3741	0.9301	0.062*	
H40C	0.1947	1.3068	0.8762	0.062*	
Cu1	0.18408 (8)	0.72680 (8)	0.82869 (7)	0.0265 (2)	
Cu2	0.34250 (8)	0.73347 (8)	0.66603 (7)	0.0249 (2)	
Cu3	0.15501 (8)	0.88478 (8)	0.66313 (7)	0.0257 (2)	
Cu4	0.33722 (8)	0.90983 (8)	0.81346 (7)	0.0276 (2)	
N1	0.1092 (6)	0.6345 (6)	0.9127 (5)	0.0299 (16)	
N2	0.1845 (8)	0.6041 (8)	1.2003 (7)	0.052 (2)	
N3	0.4330 (6)	0.6449 (7)	0.5918 (5)	0.0346 (19)	
N4	0.6961 (7)	0.5007 (7)	0.7219 (6)	0.039 (2)	
N5	0.0533 (6)	0.9531 (7)	0.5697 (5)	0.0324 (18)	
N6	−0.1965 (6)	0.8129 (7)	0.4240 (6)	0.038 (2)	
N7	0.4085 (7)	1.0081 (6)	0.8855 (6)	0.038 (2)	
N8	0.2476 (7)	1.2506 (8)	0.9930 (6)	0.046 (2)	
O1	0.2551 (5)	0.8122 (5)	0.7435 (4)	0.0287 (13)	

### Atomic displacement parameters (Å^2^)

**Table:**

	*U*^11^	*U*^22^	*U*^33^	*U*^12^	*U*^13^	*U*^23^
Br1	0.0274 (4)	0.0272 (4)	0.0235 (4)	0.0058 (3)	−0.0023 (3)	−0.0028 (3)
Br2	0.0265 (4)	0.0342 (5)	0.0260 (4)	0.0033 (4)	−0.0049 (3)	−0.0022 (4)
Br3	0.0380 (5)	0.0280 (4)	0.0250 (4)	0.0050 (4)	−0.0059 (3)	−0.0036 (4)
Br4	0.0294 (4)	0.0312 (4)	0.0222 (4)	0.0008 (4)	−0.0041 (3)	0.0007 (4)
Br5	0.0246 (4)	0.0349 (5)	0.0309 (4)	−0.0007 (4)	−0.0019 (3)	−0.0025 (4)
Br6	0.0358 (5)	0.0292 (5)	0.0346 (5)	0.0034 (4)	−0.0126 (4)	−0.0033 (4)
C1	0.034 (5)	0.028 (4)	0.012 (3)	−0.002 (4)	−0.008 (3)	−0.008 (3)
C2	0.024 (4)	0.039 (6)	0.036 (5)	−0.003 (4)	0.005 (4)	−0.012 (4)
C3	0.077 (8)	0.030 (5)	0.047 (6)	0.012 (6)	0.011 (6)	−0.022 (5)
C4	0.035 (6)	0.051 (7)	0.058 (7)	−0.016 (5)	0.008 (5)	−0.011 (6)
C5	0.024 (4)	0.032 (5)	0.036 (5)	−0.008 (4)	0.018 (4)	−0.018 (4)
C6	0.052 (6)	0.041 (6)	0.031 (5)	−0.018 (5)	0.013 (4)	0.008 (4)
C7	0.047 (6)	0.045 (6)	0.030 (5)	0.010 (5)	−0.005 (4)	0.000 (4)
C8	0.035 (5)	0.030 (5)	0.030 (4)	−0.013 (4)	0.004 (4)	0.004 (4)
C9	0.042 (6)	0.054 (7)	0.040 (5)	−0.012 (5)	−0.006 (5)	−0.005 (5)
C10	0.031 (5)	0.045 (6)	0.038 (5)	0.021 (4)	−0.007 (4)	−0.001 (4)
C11	0.027 (4)	0.061 (7)	0.016 (4)	0.005 (5)	−0.006 (3)	−0.009 (4)
C12	0.036 (5)	0.032 (5)	0.040 (5)	0.012 (4)	−0.013 (4)	−0.004 (4)
C13	0.022 (4)	0.037 (5)	0.044 (6)	0.002 (4)	0.005 (4)	−0.012 (4)
C14	0.048 (6)	0.056 (7)	0.036 (5)	0.020 (5)	0.008 (5)	−0.013 (5)
C15	0.033 (5)	0.060 (7)	0.036 (5)	0.014 (5)	0.005 (4)	−0.011 (5)
C16	0.033 (5)	0.035 (5)	0.039 (5)	0.019 (4)	−0.010 (4)	−0.013 (4)
C17	0.082 (9)	0.038 (6)	0.036 (5)	0.006 (6)	−0.008 (6)	0.024 (5)
C18	0.068 (7)	0.036 (6)	0.034 (5)	−0.029 (5)	−0.004 (5)	0.007 (4)
C19	0.044 (6)	0.037 (6)	0.062 (7)	−0.001 (5)	−0.010 (5)	0.008 (5)
C20	0.036 (6)	0.065 (8)	0.060 (7)	0.014 (6)	0.017 (5)	0.043 (6)
C21	0.041 (6)	0.056 (7)	0.046 (6)	−0.006 (5)	0.000 (5)	0.025 (5)
C22	0.044 (6)	0.058 (7)	0.033 (5)	0.014 (5)	−0.005 (5)	0.000 (5)
C23	0.032 (5)	0.039 (5)	0.034 (5)	0.013 (4)	−0.012 (4)	−0.020 (4)
C24	0.034 (5)	0.056 (7)	0.044 (6)	0.017 (5)	−0.006 (5)	0.021 (5)
C25	0.028 (5)	0.038 (5)	0.032 (4)	0.022 (4)	−0.006 (4)	−0.003 (4)
C26	0.064 (8)	0.041 (6)	0.038 (5)	0.019 (6)	−0.023 (5)	0.005 (5)
C27	0.053 (7)	0.059 (8)	0.059 (7)	0.001 (6)	0.013 (6)	−0.014 (6)
C28	0.032 (5)	0.044 (6)	0.032 (5)	−0.007 (4)	0.006 (4)	−0.012 (4)
C29	0.037 (5)	0.042 (6)	0.049 (6)	−0.008 (5)	−0.002 (4)	−0.013 (5)
C30	0.058 (7)	0.032 (6)	0.049 (6)	−0.006 (5)	−0.010 (5)	−0.002 (4)
C31	0.035 (5)	0.022 (5)	0.062 (7)	−0.003 (4)	0.008 (5)	−0.002 (5)
C32	0.051 (7)	0.030 (5)	0.048 (6)	0.015 (5)	−0.022 (5)	−0.009 (5)
C33	0.042 (6)	0.043 (6)	0.047 (6)	0.018 (5)	−0.023 (5)	−0.002 (5)
C34	0.012 (4)	0.089 (9)	0.036 (5)	−0.003 (5)	0.001 (4)	−0.015 (6)
C35	0.036 (6)	0.054 (7)	0.066 (8)	−0.014 (5)	−0.004 (5)	−0.033 (6)
C36	0.038 (5)	0.046 (6)	0.037 (5)	−0.003 (5)	−0.006 (4)	−0.012 (5)
C37	0.036 (5)	0.045 (6)	0.033 (5)	0.012 (5)	0.017 (4)	0.008 (4)
C38	0.057 (7)	0.053 (7)	0.027 (5)	−0.010 (6)	−0.009 (4)	0.011 (5)
C39	0.043 (6)	0.045 (6)	0.064 (7)	−0.017 (5)	−0.003 (5)	0.009 (6)
C40	0.041 (5)	0.053 (7)	0.033 (5)	0.014 (5)	0.018 (4)	−0.008 (4)
Cu1	0.0317 (5)	0.0242 (5)	0.0233 (5)	0.0076 (5)	−0.0020 (4)	−0.0027 (4)
Cu2	0.0231 (5)	0.0273 (5)	0.0242 (5)	0.0048 (4)	−0.0024 (4)	−0.0038 (4)
Cu3	0.0272 (5)	0.0249 (5)	0.0243 (5)	0.0030 (4)	−0.0068 (4)	−0.0012 (4)
Cu4	0.0290 (6)	0.0259 (6)	0.0273 (5)	−0.0001 (4)	−0.0066 (4)	−0.0060 (4)
N1	0.028 (4)	0.029 (4)	0.032 (4)	0.000 (3)	−0.001 (3)	0.000 (3)
N2	0.060 (6)	0.049 (6)	0.047 (5)	0.016 (5)	0.008 (5)	−0.007 (4)
N3	0.031 (4)	0.043 (5)	0.031 (4)	0.009 (4)	0.023 (3)	0.016 (3)
N4	0.041 (5)	0.044 (5)	0.030 (4)	−0.004 (4)	−0.012 (4)	0.017 (4)
N5	0.030 (4)	0.047 (5)	0.020 (3)	0.002 (4)	0.002 (3)	0.005 (3)
N6	0.031 (4)	0.048 (5)	0.037 (4)	0.012 (4)	0.004 (3)	0.011 (4)
N7	0.047 (5)	0.021 (4)	0.045 (5)	−0.001 (4)	−0.016 (4)	−0.008 (4)
N8	0.042 (5)	0.059 (6)	0.036 (4)	0.022 (4)	0.001 (4)	−0.021 (4)
O1	0.032 (3)	0.028 (3)	0.026 (3)	0.000 (2)	−0.005 (2)	−0.005 (2)

### Geometric parameters (Å, °)

**Table:**

Br1—Cu1	2.4824 (13)	C20—H20B	0.9800
Br1—Cu2	2.5565 (14)	C20—H20C	0.9800
Br2—Cu1	2.5197 (13)	C21—C22	1.316 (15)
Br2—Cu3	2.5264 (14)	C21—N5	1.321 (14)
Br3—Cu4	2.4991 (15)	C21—H21A	0.9500
Br3—Cu1	2.5212 (13)	C22—C23	1.397 (17)
Br4—Cu3	2.5097 (13)	C22—C26	1.474 (17)
Br4—Cu2	2.5178 (12)	C23—C24	1.239 (15)
Br5—Cu4	2.5162 (14)	C23—H23A	0.9500
Br5—Cu2	2.5203 (14)	C24—C25	1.419 (12)
Br6—Cu3	2.4883 (14)	C24—H24A	0.9500
Br6—Cu4	2.5066 (14)	C25—N5	1.377 (14)
C1—N1	1.348 (10)	C25—H25A	0.9500
C1—C2	1.416 (13)	C26—N6	1.561 (14)
C1—H1A	0.9500	C26—C27	1.583 (17)
C2—C3	1.430 (15)	C26—H26A	1.0000
C2—C6	1.471 (13)	C27—C28	1.625 (16)
C3—C4	1.401 (16)	C27—H27A	0.9900
C3—H3A	0.9500	C27—H27B	0.9900
C4—C5	1.371 (15)	C28—C29	1.488 (14)
C4—H4A	0.9500	C28—H28A	0.9900
C5—N1	1.406 (11)	C28—H28B	0.9900
C5—H5A	0.9500	C29—N6	1.582 (13)
C6—N2	1.453 (14)	C29—H29A	0.9900
C6—C9	1.510 (16)	C29—H29B	0.9900
C6—H6A	1.0000	C30—N6	1.399 (14)
C7—C8	1.459 (14)	C30—H30A	0.9800
C7—N2	1.503 (13)	C30—H30B	0.9800
C7—H7A	0.9900	C30—H30C	0.9800
C7—H7B	0.9900	C31—N7	1.133 (13)
C8—C9	1.486 (13)	C31—C32	1.520 (14)
C8—H8A	0.9900	C31—H31A	0.9500
C8—H8B	0.9900	C32—C33	1.285 (16)
C9—H9A	0.9900	C32—C36	1.467 (15)
C9—H9B	0.9900	C33—C34	1.358 (15)
C10—N2	1.490 (14)	C33—H33	0.9500
C10—H10A	0.9800	C34—C35	1.329 (16)
C10—H10B	0.9800	C34—H34A	0.9500
C10—H10C	0.9800	C35—N7	1.596 (14)
C11—N3	1.355 (12)	C35—H35A	0.9500
C11—C12	1.358 (14)	C36—N8	1.456 (13)
C11—H11A	0.9500	C36—C39	1.499 (15)
C12—C13	1.451 (14)	C36—H36A	1.0000
C12—C16	1.494 (12)	C37—N8	1.384 (12)
C13—C14	1.437 (15)	C37—C38	1.637 (13)
C13—H13A	0.9500	C37—H37A	0.9900
C14—C15	1.443 (14)	C37—H37B	0.9900
C14—H14A	0.9500	C38—C39	1.446 (17)
C15—N3	1.324 (12)	C38—H38A	0.9900
C15—H15A	0.9500	C38—H38B	0.9900
C16—N4	1.441 (12)	C39—H39A	0.9900
C16—C19	1.611 (16)	C39—H39B	0.9900
C16—H16A	1.0000	C40—N8	1.364 (14)
C17—N4	1.501 (11)	C40—H40A	0.9800
C17—C18	1.507 (17)	C40—H40B	0.9800
C17—H17A	0.9900	C40—H40C	0.9800
C17—H17B	0.9900	Cu1—O1	1.923 (7)
C18—C19	1.550 (14)	Cu1—N1	1.992 (8)
C18—H18A	0.9900	Cu2—O1	1.921 (6)
C18—H18B	0.9900	Cu2—N3	1.993 (8)
C19—H19A	0.9900	Cu3—O1	1.951 (6)
C19—H19B	0.9900	Cu3—N5	2.049 (7)
C20—N4	1.495 (13)	Cu4—N7	1.882 (8)
C20—H20A	0.9800	Cu4—O1	1.930 (6)
			
Cu1—Br1—Cu2	77.55 (4)	H28A—C28—H28B	108.0
Cu1—Br2—Cu3	77.83 (4)	C28—C29—N6	103.2 (7)
Cu4—Br3—Cu1	77.74 (4)	C28—C29—H29A	111.1
Cu3—Br4—Cu2	77.63 (4)	N6—C29—H29A	111.1
Cu4—Br5—Cu2	77.43 (4)	C28—C29—H29B	111.1
Cu3—Br6—Cu4	77.99 (4)	N6—C29—H29B	111.1
N1—C1—C2	121.4 (8)	H29A—C29—H29B	109.1
N1—C1—H1A	119.3	N6—C30—H30A	109.5
C2—C1—H1A	119.3	N6—C30—H30B	109.5
C1—C2—C3	117.7 (9)	H30A—C30—H30B	109.5
C1—C2—C6	117.0 (8)	N6—C30—H30C	109.5
C3—C2—C6	123.5 (9)	H30A—C30—H30C	109.5
C4—C3—C2	119.6 (11)	H30B—C30—H30C	109.5
C4—C3—H3A	120.2	N7—C31—C32	134.8 (10)
C2—C3—H3A	120.2	N7—C31—H31A	112.6
C5—C4—C3	118.9 (10)	C32—C31—H31A	112.6
C5—C4—H4A	120.6	C33—C32—C36	130.6 (10)
C3—C4—H4A	120.6	C33—C32—C31	107.5 (10)
C4—C5—N1	121.9 (9)	C36—C32—C31	121.3 (10)
C4—C5—H5A	119.1	C32—C33—C34	127.2 (11)
N1—C5—H5A	119.1	C32—C33—H33	116.4
N2—C6—C2	111.8 (9)	C34—C33—H33	116.4
N2—C6—C9	103.0 (8)	C35—C34—C33	119.5 (10)
C2—C6—C9	117.1 (9)	C35—C34—H34A	120.3
N2—C6—H6A	108.2	C33—C34—H34A	120.3
C2—C6—H6A	108.2	C34—C35—N7	116.2 (10)
C9—C6—H6A	108.2	C34—C35—H35A	121.9
C8—C7—N2	101.4 (8)	N7—C35—H35A	121.9
C8—C7—H7A	111.5	N8—C36—C32	117.6 (8)
N2—C7—H7A	111.5	N8—C36—C39	100.9 (9)
C8—C7—H7B	111.5	C32—C36—C39	115.6 (10)
N2—C7—H7B	111.5	N8—C36—H36A	107.4
H7A—C7—H7B	109.3	C32—C36—H36A	107.4
C7—C8—C9	111.3 (9)	C39—C36—H36A	107.4
C7—C8—H8A	109.4	N8—C37—C38	101.3 (8)
C9—C8—H8A	109.4	N8—C37—H37A	111.5
C7—C8—H8B	109.4	C38—C37—H37A	111.5
C9—C8—H8B	109.4	N8—C37—H37B	111.5
H8A—C8—H8B	108.0	C38—C37—H37B	111.5
C8—C9—C6	101.5 (9)	H37A—C37—H37B	109.3
C8—C9—H9A	111.5	C39—C38—C37	104.7 (8)
C6—C9—H9A	111.5	C39—C38—H38A	110.8
C8—C9—H9B	111.5	C37—C38—H38A	110.8
C6—C9—H9B	111.5	C39—C38—H38B	110.8
H9A—C9—H9B	109.3	C37—C38—H38B	110.8
N2—C10—H10A	109.5	H38A—C38—H38B	108.9
N2—C10—H10B	109.5	C38—C39—C36	108.7 (10)
H10A—C10—H10B	109.5	C38—C39—H39A	110.0
N2—C10—H10C	109.5	C36—C39—H39A	110.0
H10A—C10—H10C	109.5	C38—C39—H39B	110.0
H10B—C10—H10C	109.5	C36—C39—H39B	110.0
N3—C11—C12	120.3 (8)	H39A—C39—H39B	108.3
N3—C11—H11A	119.9	N8—C40—H40A	109.5
C12—C11—H11A	119.9	N8—C40—H40B	109.5
C11—C12—C13	126.6 (8)	H40A—C40—H40B	109.5
C11—C12—C16	118.8 (9)	N8—C40—H40C	109.5
C13—C12—C16	114.5 (9)	H40A—C40—H40C	109.5
C14—C13—C12	111.1 (8)	H40B—C40—H40C	109.5
C14—C13—H13A	124.4	O1—Cu1—N1	177.7 (3)
C12—C13—H13A	124.4	O1—Cu1—Br1	86.9 (2)
C13—C14—C15	118.7 (9)	N1—Cu1—Br1	91.6 (2)
C13—C14—H14A	120.6	O1—Cu1—Br2	86.56 (19)
C15—C14—H14A	120.6	N1—Cu1—Br2	92.9 (2)
N3—C15—C14	125.4 (10)	Br1—Cu1—Br2	125.77 (5)
N3—C15—H15A	117.3	O1—Cu1—Br3	85.83 (18)
C14—C15—H15A	117.3	N1—Cu1—Br3	96.5 (2)
N4—C16—C12	113.6 (8)	Br1—Cu1—Br3	121.13 (5)
N4—C16—C19	103.1 (8)	Br2—Cu1—Br3	111.94 (5)
C12—C16—C19	114.3 (8)	O1—Cu2—N3	176.4 (3)
N4—C16—H16A	108.5	O1—Cu2—Br4	86.60 (18)
C12—C16—H16A	108.5	N3—Cu2—Br4	96.0 (2)
C19—C16—H16A	108.5	O1—Cu2—Br5	86.45 (19)
N4—C17—C18	102.2 (9)	N3—Cu2—Br5	94.3 (3)
N4—C17—H17A	111.3	Br4—Cu2—Br5	123.46 (6)
C18—C17—H17A	111.3	O1—Cu2—Br1	84.8 (2)
N4—C17—H17B	111.3	N3—Cu2—Br1	91.7 (2)
C18—C17—H17B	111.3	Br4—Cu2—Br1	118.39 (5)
H17A—C17—H17B	109.2	Br5—Cu2—Br1	116.69 (5)
C17—C18—C19	106.7 (8)	O1—Cu3—N5	175.1 (3)
C17—C18—H18A	110.4	O1—Cu3—Br6	86.91 (19)
C19—C18—H18A	110.4	N5—Cu3—Br6	96.3 (2)
C17—C18—H18B	110.4	O1—Cu3—Br4	86.20 (19)
C19—C18—H18B	110.4	N5—Cu3—Br4	89.0 (2)
H18A—C18—H18B	108.6	Br6—Cu3—Br4	122.88 (5)
C18—C19—C16	104.1 (8)	O1—Cu3—Br2	85.78 (19)
C18—C19—H19A	110.9	N5—Cu3—Br2	95.7 (2)
C16—C19—H19A	110.9	Br6—Cu3—Br2	121.51 (5)
C18—C19—H19B	110.9	Br4—Cu3—Br2	114.38 (5)
C16—C19—H19B	110.9	N7—Cu4—O1	175.9 (4)
H19A—C19—H19B	109.0	N7—Cu4—Br3	91.2 (3)
N4—C20—H20A	109.5	O1—Cu4—Br3	86.29 (19)
N4—C20—H20B	109.5	N7—Cu4—Br6	91.4 (2)
H20A—C20—H20B	109.5	O1—Cu4—Br6	86.83 (19)
N4—C20—H20C	109.5	Br3—Cu4—Br6	116.56 (5)
H20A—C20—H20C	109.5	N7—Cu4—Br5	97.7 (3)
H20B—C20—H20C	109.5	O1—Cu4—Br5	86.36 (19)
C22—C21—N5	123.1 (11)	Br3—Cu4—Br5	128.08 (5)
C22—C21—H21A	118.5	Br6—Cu4—Br5	114.23 (5)
N5—C21—H21A	118.5	C1—N1—C5	119.4 (8)
C21—C22—C23	117.8 (11)	C1—N1—Cu1	122.0 (6)
C21—C22—C26	124.5 (12)	C5—N1—Cu1	118.6 (6)
C23—C22—C26	117.7 (9)	C6—N2—C10	105.4 (9)
C24—C23—C22	120.6 (10)	C6—N2—C7	104.8 (9)
C24—C23—H23A	119.7	C10—N2—C7	106.9 (8)
C22—C23—H23A	119.7	C15—N3—C11	117.5 (8)
C23—C24—C25	123.0 (11)	C15—N3—Cu2	125.7 (7)
C23—C24—H24A	118.5	C11—N3—Cu2	116.8 (6)
C25—C24—H24A	118.5	C16—N4—C20	116.8 (9)
N5—C25—C24	115.4 (9)	C16—N4—C17	108.7 (8)
N5—C25—H25A	122.3	C20—N4—C17	112.0 (8)
C24—C25—H25A	122.3	C21—N5—C25	119.7 (8)
C22—C26—N6	106.4 (9)	C21—N5—Cu3	121.1 (7)
C22—C26—C27	112.9 (9)	C25—N5—Cu3	118.9 (6)
N6—C26—C27	103.9 (9)	C30—N6—C26	114.2 (8)
C22—C26—H26A	111.1	C30—N6—C29	106.9 (8)
N6—C26—H26A	111.1	C26—N6—C29	100.3 (8)
C27—C26—H26A	111.1	C31—N7—C35	111.7 (8)
C26—C27—C28	98.1 (9)	C31—N7—Cu4	126.9 (8)
C26—C27—H27A	112.1	C35—N7—Cu4	121.3 (6)
C28—C27—H27A	112.1	C40—N8—C37	120.6 (9)
C26—C27—H27B	112.1	C40—N8—C36	110.2 (9)
C28—C27—H27B	112.1	C37—N8—C36	116.1 (8)
H27A—C27—H27B	109.8	Cu2—O1—Cu1	110.4 (3)
C29—C28—C27	111.1 (9)	Cu2—O1—Cu4	109.7 (3)
C29—C28—H28A	109.4	Cu1—O1—Cu4	109.7 (3)
C27—C28—H28A	109.4	Cu2—O1—Cu3	109.0 (3)
C29—C28—H28B	109.4	Cu1—O1—Cu3	109.8 (3)
C27—C28—H28B	109.4	Cu4—O1—Cu3	108.2 (3)

### Hydrogen-bond geometry (Å, °)

**Table:**

*D*—H···*A*	*D*—H	H···*A*	*D*···*A*	*D*—H···*A*
C1—H1A···Br3	0.95	2.60	3.292 (9)	130
C15—H15A···Br4	0.95	2.71	3.362 (10)	126
C21—H21A···Br2	0.95	2.77	3.372 (10)	122
C25—H25A···Br6	0.95	2.75	3.332 (9)	120
C30—H30C···Br6^i^	0.98	2.92	3.844 (10)	158
C35—H35A···Br5	0.95	2.68	3.259 (10)	120
C39—H39B···Br5^ii^	0.99	2.88	3.764 (11)	150

Symmetry codes: (i) −*x*, *y*−1/2, −*z*+1; (ii) −*x*+1, *y*+1/2, −*z*+2.
